# *DICER1* alterations in thyroid lesions: a systematic review and meta-analysis with clinicopathologic implications

**DOI:** 10.1007/s00428-026-04580-5

**Published:** 2026-05-28

**Authors:** Patrizia Straccia, Vincenzo Fiorentino, Alessia Piermattei, Antonino Mulè, Esther Diana Rossi, Esther Rossi

**Affiliations:** 1 Division of Anatomic Pathology and Histology, Fondazione Policlinico “Agostino Gemelli”-IRCCS, Università Cattolica del Sacro Cuore, Largo Francesco Vito, 1, Rome, 00168 Italy; 2 Anatomic Pathology Unit, Department of Human Pathology in Adult and Developmental Age “Gaetano Barresi’’, University of Messina, Messina, 98125 Italy

**Keywords:** *DICER1*, Thyroid lesions, Thyroid nodules, Thyroid neoplasms, Molecular pathology

## Abstract

*DICER1* is a key RNase III endoribonuclease involved in microRNA biogenesis and is implicated in both constitutional DICER1-related tumor predisposition and sporadic thyroid tumorigenesis. In thyroid pathology, *DICER1* alterations have been reported across a broad spectrum of lesions, but their reported frequency and clinicopathologic significance vary substantially across published series. We performed a systematic review of thyroid-focused studies published between 2020 and 2025. Studies were included in the quantitative synthesis only if they provided an interpretable lesion-level numerator and denominator for thyroid lesions harbouring *DICER1* alterations. Additional thyroid-focused studies that refined the cytomorphologic, histologic, and lesion-spectrum interpretation of *DICER1*-associated thyroid disease, but lacked a suitable denominator for prevalence meta-analysis, were retained for qualitative clinicopathologic synthesis. Random-effects meta-analysis of logit-transformed proportions was used for the quantitative component. Seven studies met criteria for quantitative synthesis, comprising 16,831 thyroid lesions, of which 317 were reported as *DICER1*-altered. Study-level proportions ranged from 1.4% in a large adult consecutive molecular-testing cohort to 22.0% in a pediatric follicular-patterned tumor cohort. The pooled proportion of thyroid lesions harbouring *DICER1* alterations was 5.76% (95% CI, 2.49%-12.78%), with substantial heterogeneity (I² = 96.94%). Exploratory stratification showed lower pooled proportions in predominantly adult cohorts and higher pooled proportions in pediatric or young-enriched cohorts. Qualitative synthesis showed that *DICER1* alterations occur across a wide thyroid lesion spectrum, including multinodular and follicular nodular disease, follicular adenoma, NIFTP, follicular thyroid carcinoma, follicular-patterned papillary thyroid carcinoma, and rare higher-grade or primitive malignant tumors such as thyroblastoma. Cytologically and histologically, these lesions frequently show follicular-patterned architecture, often with macrofollicular or mixed follicular growth and relatively bland nuclear features.Seven studies met criteria for quantitative synthesis, comprising 16,831 thyroid lesions, of which 317 were reported as *DICER1*-altered. Study-level proportions ranged from 1.4% in a large adult consecutive molecular-testing cohort to 22.0% in a pediatric follicular-patterned tumor cohort. The pooled proportion of thyroid lesions harbouring *DICER1* alterations was 5.76% (95% CI, 2.49%-12.78%), with substantial heterogeneity (I² = 96.94%). Exploratory stratification showed lower pooled proportions in predominantly adult cohorts and higher pooled proportions in pediatric or young-enriched cohorts. Qualitative synthesis showed that *DICER1* alterations occur across a wide thyroid lesion spectrum, including multinodular and follicular nodular disease, follicular adenoma, NIFTP, follicular thyroid carcinoma, follicular-patterned papillary thyroid carcinoma, and rare higher-grade or primitive malignant tumors such as thyroblastoma. Cytologically and histologically, these lesions frequently show follicular-patterned architecture, often with macrofollicular or mixed follicular growth and relatively bland nuclear features. *DICER1* alterations are uncommon in large unselected adult thyroid cohorts but enriched in pediatric, young-adult, and follicular-patterned settings. They should not be interpreted as isolated lesion-specific markers. Rather, their diagnostic significance lies in the integration of molecular findings with lesion type, cytomorphology, histology, patient age, and clinical context, including the selective recognition of cases in which constitutional *DICER1***-**related tumor predisposition should be considered.

**Supplementary Information:**

The online version contains supplementary material available at 10.1007/s00428-026-04580-5.

## Introduction

Thyroid nodules represent one of the most common diagnostic challenges in surgical pathology and cytopathology, with prevalence estimates reaching up to 60% in the adult population when assessed by high-resolution ultrasonography [1, 2]. Despite this high prevalence, only a small fraction of nodules corresponds to malignant neoplasms, creating an inherent tension between overdiagnosis and underdiagnosis. Fine-needle aspiration cytology (FNAC) remains the cornerstone of preoperative evaluation, yet indeterminate cytologic categories continue to represent a major area of uncertainty in daily practice [3–7]. Over the past decade, the incorporation of molecular testing into the evaluation of thyroid nodules has substantially changed this landscape by refining risk stratification, reducing unnecessary surgery in some settings, and providing biologic insight into lesions that cannot be fully classified on morphology alone. Molecular panels initially focused on canonical driver alterations such as *BRAF*, *RAS*, *RET* and *PAX8–PPARG* rearrangements, but progressively expanded to include a broader spectrum of genes involved in chromatin remodeling, RNA processing and transcriptional regulation [8–13].

Within this evolving landscape, *DICER1* has emerged as a gene of particular interest, not only because of its role in thyroid tumorigenesis but also due to its association with a multisystem tumor predisposition syndrome [14–16]. *DICER1* encodes a cytoplasmic RNase III endoribonuclease that is essential for the final steps of microRNA biogenesis, processing precursor microRNAs into mature duplexes that regulate post-transcriptional gene expression [16, 17]. Alterations affecting *DICER1*, especially recurrent mutations involving the RNase III domains, perturb microRNA processing rather than simply abolishing protein function, resulting in a distinctive biologic state characterized by imbalance of 5p- and 3p-derived microRNAs, altered differentiation programs, and proliferative signaling. Experimental and mechanistic studies have further shown that *DICER1* dysregulation is relevant to thyroid-cell biology itself, affecting proliferation, differentiation, and microRNA machinery in thyroid models [18, 19].

The clinical importance of *DICER1* was first recognized in the context of hereditary tumor predisposition. Germline pathogenic variants underlie what is now more appropriately termed *DICER1*-related tumor predisposition, a pleiotropic disorder associated with pleuropulmonary blastoma, cystic nephroma, ovarian Sertoli-Leydig cell tumor, and a broad range of additional neoplastic and dysplastic conditions [14, 15]. Thyroid disease is among the most frequent manifestations of this predisposition and may include multinodular goiter, follicular-patterned proliferations, and differentiated thyroid carcinoma, often presenting at a relatively young age [20, 21]. In some individuals, thyroid disease may be the first or only clinically apparent manifestation, emphasizing the potential diagnostic significance of recognizing *DICER1*-associated thyroid lesions in routine pathologic practice [22].

Beyond the constitutional setting, somatic *DICER1* alterations have increasingly been documented in sporadic thyroid lesions. The currently available thyroid literature indicates that these alterations are particularly enriched in pediatric and young-adult cohorts and in follicular-patterned lesions. Across published studies, *DICER1* has been described in multinodular and follicular nodular disease, follicular adenoma, follicular thyroid carcinoma, follicular-patterned papillary thyroid carcinoma, and, in more limited series, poorly differentiated carcinoma and other unusual high-grade or primitive malignant thyroid tumors [23, 24]. Several studies also suggest recurring morphologic and cytomorphologic associations, including follicular-patterned architecture, macrofollicular or mixed follicular growth, relatively bland nuclear features, and multinodular background, although none of these findings is sufficiently specific to define *DICER1* status in the absence of molecular analysis [22, 24].

At the same time, the reported frequency of *DICER1* alterations in thyroid lesions varies strikingly across studies. Large routine-practice adult cohorts generally report low frequencies, whereas pediatric, young-adult, and histotype-enriched cohorts often report substantially higher proportions. This variability likely reflects real differences in age distribution, lesion selection, specimen type, and molecular methodology, but it also complicates the interpretation of isolated numeric estimates [20, 25]. In addition, not all thyroid-focused *DICER1* studies address the same question: some are suited to estimating study-level proportions, whereas others are more informative for cytomorphology, histology, or lesion-spectrum characterization [26, 27]. Hence, a systematic synthesis that explicitly separates these analytic purposes is needed.

We therefore performed a systematic review of thyroid-focused studies published between 2020 and 2025, with a dual objective. First, we sought to quantitatively summarize the reported frequency of *DICER1* alterations using only studies that provided interpretable lesion-level numerators and denominators. Second, we aimed to qualitatively define the clinicopathologic spectrum of *DICER1*-associated thyroid disease by incorporating thyroid-focused studies that, although unsuitable for prevalence meta-analysis, substantially refine the cytologic, histologic, and lesion-specific interpretation of these alterations. By integrating both quantitative and qualitative evidence, the present study aims to provide a more clinically and pathologically meaningful framework for interpreting *DICER1* alterations in thyroid lesions.

## Materials and methods

### Study design

This study was conducted as a systematic review and meta-analysis aimed at synthesizing contemporary evidence on the frequency and clinicopathologic significance of *DICER1* alterations in thyroid lesions. Given the marked heterogeneity of the available literature, the review was structured a priori into two components: a quantitative synthesis, restricted to studies providing an interpretable lesion-level numerator and denominator for thyroid lesions harbouring *DICER1* alterations, and a qualitative clinicopathologic synthesis, including thyroid-focused studies that were informative for morphology, cytology, lesion spectrum, or genotype-phenotype correlation but were not suitable for prevalence meta-analysis because of molecular or morphologic enrichment at cohort entry. The overall analytic framework was based on study-level proportions of *DICER1*-altered thyroid lesions and incorporated a stricter distinction between prevalence-eligible studies and studies retained for qualitative clinicopathologic synthesis only.

### Literature search strategy

A literature search was performed to identify original studies published between January 1, 2020 and December 31, 2025 reporting thyroid-specific *DICER1* findings. This time window was selected to capture the contemporary molecular pathology era, in which next-generation sequencing and thyroid-focused molecular classifiers became more widely integrated into routine cytologic and histologic practice. The electronic search strategy included the terms *thyroid*, *thyroid nodule*, *thyroid neoplasm*, *thyroid carcinoma*, *thyroid cancer*, and *DICER1*, used alone and in combination. Reference lists of relevant studies were also screened manually to identify additional thyroid-focused original reports.

### Study selection and classification

Full-text review was used to determine whether each study was suitable for quantitative pooling, qualitative clinicopathologic synthesis only, or background discussion. Studies were included in the quantitative synthesis if they reported original thyroid-specific data and provided both:

1. a numerator, defined as the number of thyroid lesions reported as harbouring DICER1 alterations; and
2. a denominator, defined as the total number of thyroid lesions analyzed within that study cohort.

On this basis, seven studies were included in the quantitative synthesis: Chong et al. [28], Onder et al. [29], Minna et al. [30], Wang et al. [31], Mastnikova et al. [32], Lee et al. [33], and Bae et al. [34] These studies comprised adult routine-practice cohorts, pediatric/young cohorts, and follicular-patterned tumor cohorts, but all provided interpretable lesion-level numerators and denominators.

Thyroid-focused studies were retained for qualitative clinicopathologic synthesis only when they described selected *DICER1*-positive or morphology-enriched cohorts without a denominator suitable for prevalence meta-analysis. This applied in particular to studies centred on preselected DICER1-mutant FNAs or on thyroid nodules already defined by molecular criteria. This category included Darbinyan et al. [35], Lengyel et al. [36], Jitpasutham et al. [37], Karimkhan et al. [38], Miranda et al. [39], and Wu et al. [40].

Studies were not included in the formal systematic-review dataset if they were non-thyroid lesion-level, germline/population-based rather than lesion-based, or primarily mechanistic/structural.

The literature search identified 163 records across PubMed, Scopus, and Web of Science. After removal of 62 duplicates, 101 records underwent title and abstract screening, 26 full-text reports were assessed for eligibility, and 13 studies were ultimately included in the systematic review, including 7 in the quantitative synthesis and 6 in the qualitative clinicopathologic synthesis (Supplementary Fig. 1). The characteristics of the studies included in the quantitative and qualitative syntheses are summarized in Tables 1 and 2, respectively.

**Table 1:** Quantitative studies included in the meta-analysis of *DICER1* alterations in thyroid lesions

Study	Study design / setting	Thyroid lesions analyzed (*n*)	DICER1-altered lesions (*n*)	Proportion (%)	Age setting	Specimen type	Main cohort / lesion context	Molecular method	Key clinicopathologic note
**Chong et al.**,** 2021** [28]	Population-based consecutive cohort	14,993	214	1.4	Predominantly adult	FNA	Adult-onset thyroid nodules with indeterminate cytology	ThyroSeq v3; full *DICER1* sequencing in selected cases	Largest routine-practice cohort; hotspot-positive nodules frequently showed second-hit *DICER1* events and relative mutual exclusivity with other thyroid drivers
**Onder et al.**,** 2022** [29]	Institutional retrospective cohort	56	8	14.3	Pediatric	Resection histology	Pediatric papillary thyroid carcinoma	Hotspot *DICER1* mutation analysis	DICER1-mutant tumors were enriched in low-risk follicular-patterned PPTCs, especially invasive encapsulated FVPTC
**Minna et al.**,** 2023** [30]	Institutional retrospective molecular series	30	2	6.7	Adult	Resection histology	Follicular cell-derived thyroid tumors, largely follicular-patterned and *RAS*-like	Whole-exome sequencing and targeted RNA-seq	DICER1-mutant adult tumors occurred in a follicular-patterned/RAS-like molecular context
**Wang et al.**,** 2025** [31]	Consecutive single-center cohort	899	52	5.8	Predominantly adult	FNA	Bethesda II/III/IV thyroid nodules	Sanger sequencing for *DICER1* exons 24–25; BRAFV600E testing in Bethesda III/IV	*DICER1* and *BRAF*V600E were mutually exclusive in Bethesda III/IV nodules
**Mastnikova et al.**,** 2024** [32]	Retrospective pediatric / young-adult cohort	350	24	6.9	Pediatric and young adult	Mixed postoperative tissue and FNAB	Thyroid nodules in patients aged 2–21 years	NGS plus MLPA; germline testing in *DICER1*-positive cases	Germline variants were identified in a substantial subset; *DICER1*-positive carcinomas showed low-invasiveness features
**Lee et al.**,** 2022** [17]	Institutional consecutive FNA cohort with molecular testing	462	8	1.7	Adult	FNA	Thyroid nodules with available ThyroSeq v3	ThyroSeq v3	*DICER1*-mutant nodules occurred in younger adults, often with bland cytomorphology and benign resection outcomes when available
**Bae et al.**,** 2021** [34]	Two-institution pediatric tumor cohort	41	9	22.0	Pediatric	Resection histology	Pediatric follicular-patterned thyroid tumors	Targeted deep sequencing of 49 genes plus fusion panel	*DICER1* was the most frequent genetic alteration and was identified across FA, FTC, and NIFTP-like lesions

Abbreviations: *FA* follicular adenoma; *FNA* fine-needle aspiration; *FNAB* fine-needle aspiration biopsy; *FTC* follicular thyroid carcinoma; *FVPTC* follicular variant papillary thyroid carcinoma; *MLPA* multiplex ligation-dependent probe amplification; *NGS* next-generation sequencing; *NIFTP* noninvasive follicular thyroid neoplasm with papillary-like nuclear features; *PPTC* pediatric papillary thyroid carcinoma

Only studies providing interpretable lesion-level numerators and denominators were included in the quantitative synthesis. Studies describing molecularly selected DICER1-positive cohorts or morphology-enriched cohorts without a prevalence denominator were retained for qualitative clinicopathologic synthesis only

**Table 2:** Thyroid-focused studies included in the qualitative clinicopathologic synthesis only

Study	Study design / setting	Cohort described	Main contribution to the review	Why not included in the quantitative meta-analysis
**Darbinyan et al.**,** 2020** [35]	Cytology–histopathology correlation study	7 thyroid FNA cases from patients with *DICER1* mutation	Defines early cytomorphologic patterns of DICER1-associated thyroid disease; shows follicular-derived lesions with scant colloid, moderate cellularity, microfollicles, and histologic confirmation of neoplasia in 6/7 cases, including FC, PTC, and PDTC	No denominator of all thyroid lesions tested for *DICER1*; cohort starts from already identified *DICER1*-mutated cases
**Lengyel et al.**,** 2024** [36]	Multipractice FNA cohort	18 *DICER1*-altered thyroid FNAs from 17 patients	Strongest cytomorphologic/histologic refinement of *DICER1*-altered thyroid lesions on FNA; highlights mixed macro-/microfollicular, *RAS*-like bland cytology, and broad resection spectrum from FA to high-grade follicular-derived non-anaplastic carcinoma	Molecularly selected *DICER1*-altered cohort; denominator is not all lesions tested, but only *DICER1*-positive FNAs
**Jitpasutham et al.**,** 2024** [37]	Comparative FNA cohort from two academic institutions	117 thyroid FNAs with either *DICER1* mutation or *PTEN* alteration; 36 had *DICER1* mutation	Useful for cytology, multinodular background, and resection spectrum; shows that *DICER1*-mutant FNAs are mostly AUS/FN, frequently microfollicular, and can include entities such as NIFTP, PTC, and thyroblastoma	Denominator is a preselected molecular cohort (*DICER1* or *PTEN* altered), not all thyroid lesions analyzed for *DICER1* prevalence
**Karimkhan et al.**,** 2025** [38]	Multicenter observational study	88 patients with somatic *DICER1* mutations in Bethesda III/IV thyroid cytology samples	Important for adult indeterminate nodules, malignancy rate among *DICER1*-positive cases, hotspot profile, and outcome interpretation in Bethesda III/IV context	The study explicitly included only patients with *DICER1*-mutated nodules; no prevalence denominator for all tested Bethesda III/IV lesions
**Miranda et al.**,** 2024** [39]	Retrospective multinodular goiter study	154 patients with large MNG evaluated; 17 had *DICER1* variants	Useful for discussing multinodular goiter and benign *DICER1*-related thyroid disease; highlights germline/synonymous variants in large goiters and the absence of a second-hit pattern in most cases	Not comparable with the oncologic/hotspot prevalence studies: most variants were synonymous and likely benign, with no second-hit somatic mutation in most cases
**Wu et al.**,** 2025** [40]	Retrospective cytopathology study	15 *DICER1*-mutated thyroid tumors identified among 163 histologically suspected *DICER1*-related tumors	Useful for cytopathologic phenotype: macrofollicles, small round dark nuclei, mostly benign/indeterminate preoperative categories, and mixed benign-borderline-malignant resection outcomes	Morphology-enriched cohort rather than an unselected lesion-level prevalence series

Abbreviations: *AUS* atypia of undetermined significance; *FA* follicular adenoma; *FC* follicular carcinoma; *FNA* fine-needle aspiration; *MNG* multinodular goiter; *NIFTP* noninvasive follicular thyroid neoplasm with papillary-like nuclear features; *PDTC* poorly differentiated thyroid carcinoma; *PTC* papillary thyroid carcinoma

These studies were retained for qualitative clinicopathologic synthesis because they refined the cytomorphologic, histologic, and lesion-spectrum interpretation of DICER1-associated thyroid disease, but were not suitable for prevalence meta-analysis owing to molecular preselection, morphology enrichment, lack of an interpretable lesion-level denominator, or non-comparable variant definitions

### Eligibility criteria

Eligible quantitative studies were required to meet all of the following criteria:

1. original research;
2. thyroid-specific data;
3. publication within the predefined 2020–2025 window;
4. extractable lesion-level numerator and denominator;
5. sufficient methodological detail to identify the cohort context, specimen type, and molecular approach.

Both cytologic and histologic cohorts were eligible. Studies including benign, borderline, or malignant thyroid lesions were all considered eligible, provided the denominator represented the number of lesions actually analyzed for *DICER1* within a defined study cohort. Studies were excluded from the quantitative synthesis if thyroid lesions were not analyzed separately from other tumor types, if only *DICER1*-positive lesions were described without a prevalence denominator, or if the reported alterations consisted predominantly of benign germline or synonymous variation rather than pathogenic or hotspot lesion-level alterations. This consideration was particularly relevant for certain multinodular goitre studies in which the molecular findings were not directly comparable with the hotspot-driven thyroid lesion cohorts included in the pooled analysis.

## Data extraction

Data were extracted using a standardized study-level approach. For each study, the following variables were recorded: first author and year of publication; study design and institutional setting; number of thyroid lesions analyzed; number of *DICER1*-altered lesions; age setting (predominantly adult, pediatric, young adult, or mixed); specimen type (fine-needle aspiration, resection histology, or mixed); principal lesion categories included; and molecular testing methodology.

Because the operational definition of a *DICER1*-altered lesion was not fully uniform across studies, *DICER1* status was recorded according to the original source publication. When available, hotspot mutations, pathogenic or likely pathogenic variants, variants of uncertain significance, and germline versus somatic status were documented separately. Molecular methods varied substantially and included targeted hotspot sequencing, ThyroSeq-based next-generation sequencing, broad tumor panels, full gene sequencing, and multiplex ligation-dependent probe amplification (MLPA)-based copy-number evaluation. These variables were used to support both the quantitative study-level summary (Table 1) and the qualitative clinicopathologic synthesis (Table 2).

### Outcome definition

The primary quantitative outcome was the study-level proportion of thyroid lesions reported as harbouring DICER1 alterations. This was defined as:

$$\:\mathrm{Study-level\:proportion}=\frac{\mathrm{Number\:of\:thyroid\:lesions\:reported\:as\:}\mathrm{DICER1}\mathrm{-altered}}{\mathrm{Total\:number\:of\:thyroid\:lesions\:analyzed}}$$

This outcome was interpreted strictly as a cohort-level frequency measure and not as a surrogate for malignancy risk, progression, or direct clinical outcome. Given the substantial differences in age setting, lesion composition, specimen type, and molecular testing strategies, pooled estimates were interpreted as summary measures of heterogeneous study-level proportions rather than as a single universally applicable prevalence value.

### Quantitative synthesis and statistical analysis

For each study included in the quantitative synthesis, the study-specific proportion of *DICER1*-altered thyroid lesions was calculated directly from the reported numerator and denominator. Meta-analysis of proportions was then performed using a random-effects model, because substantial between-study heterogeneity was anticipated on clinical and methodological grounds. Proportions were logit-transformed before pooling, and pooled estimates were calculated using inverse-variance weighting. A continuity correction was prespecified for studies with zero events or complete event rates, although it was not required in the final 7-study quantitative dataset because no study reported a proportion of 0 or 1.

Between-study heterogeneity was assessed using Cochran’s Q statistic and quantified with the I² statistic. Between-study variance was summarized using τ² and τ. Because the available studies represented biologically and clinically distinct populations, the pooled estimate was interpreted cautiously and always in conjunction with the individual study settings.

### Sensitivity and exploratory analyses

A leave-one-out sensitivity analysis was performed by repeating the random-effects meta-analysis after sequential exclusion of each study to assess the stability of the pooled estimate. In addition, a prespecified exploratory descriptive stratification by age setting was undertaken to compare predominantly adult cohorts with pediatric/young or pediatric-enriched cohorts, given the repeated observation across the literature that *DICER1* alterations are enriched in younger patients and in pediatric follicular-patterned lesions. These subgroup analyses were considered exploratory and hypothesis-generating rather than confirmatory.

### Assessment of small-study effects

Potential small-study effects were explored using Egger’s regression test. Because the number of quantitatively pooled studies was limited and clinical heterogeneity was substantial, this analysis was interpreted cautiously, recognizing that asymmetry may reflect true differences in study design and cohort enrichment rather than publication bias alone.

### Use of qualitative evidence

Studies not suitable for quantitative pooling were synthesized qualitatively to refine the clinicopathologic interpretation of *DICER1*-altered thyroid lesions. These studies were especially informative for cytomorphology, histologic spectrum, molecularly enriched lesion profiles, and the recognition of rare entities such as thyroblastoma or high-grade thyroid carcinoma associated with *DICER1* alterations. Qualitative evidence was not incorporated into the pooled estimate but was used to contextualize the numeric findings and to support the lesion-specific interpretation of the literature.

## Results

### Quantitative synthesis

Seven studies met the predefined criteria for quantitative synthesis because they provided interpretable lesion-level numerators and denominators for thyroid lesions harbouring *DICER1* alterations. Collectively, these studies included 16,831 thyroid lesions, of which 317 were reported as *DICER1*-altered, corresponding to a crude aggregated proportion of 1.88%. The study-level frequencies varied substantially across clinical settings. The lowest proportion was observed in the large adult consecutive ThyroSeq-based cohort reported by Chong et al. (214/14,993; 1.4%) [28], whereas higher proportions were observed in pediatric, young-adult, or histotype-enriched cohorts, including Onder et al. (8/56; 14.3%) [29] Minna et al. (2/30; 6.7%) [30] Wang et al. (52/899; 5.8%) [31], Mastnikova et al. (24/350; 6.9%) [32], Lee et al. (8/462; 1.7%) [33], and Bae et al. (9/41; 22.0%) [34]. No study reported a proportion of 0 or 1; therefore, no continuity correction was required in the final quantitative model. The principal characteristics of the seven quantitatively pooled studies are summarized in Table 1.

**Fig. 1:**
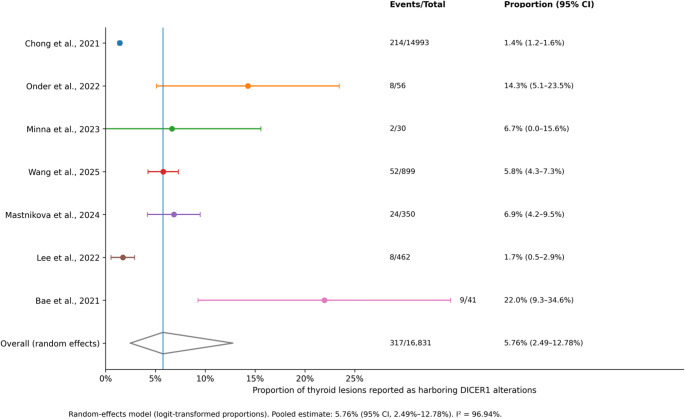
Forest plot of study-level proportions of thyroid lesions reported as harbouring *DICER1* alterations. Squares represent study-specific estimates weighted by inverse variance; horizontal lines indicate 95% confidence intervals; the diamond represents the pooled random-effects estimate

Random-effects meta-analysis of logit-transformed study proportions yielded a pooled estimate of 5.76% (95% CI, 2.49%-12.78%), with substantial between-study heterogeneity (Q = 196.34; I² = 96.94%; τ² = 1.2653; τ = 1.1249). These findings indicate that the overall pooled proportion should be interpreted as a summary of highly heterogeneous study populations rather than as a universally applicable prevalence value. As expected, inverse-variance random-effects weighting reduced the numerical dominance of the largest adult cohorts, resulting in relatively balanced study weights across the 7-study model. The corresponding forest plot should therefore be interpreted primarily as a visualization of heterogeneity across clinically distinct study settings. The study-level proportions and pooled random-effects estimate are illustrated in Fig. 1.

Leave-one-out sensitivity analysis did not identify a single dominant study driving the overall result, with pooled estimates remaining within a relatively narrow range, from 4.52% after exclusion of Bae et al. to 7.34% after exclusion of Chong et al. [28, 34] Exploratory stratification by age setting further supported the clinical interpretation of the dataset. In predominantly adult cohorts [17, 28, 30, 31], the pooled proportion was 2.95% (95% CI, 1.13%-7.52%; I² = 96.52%), whereas in pediatric or young-enriched cohorts [17, 29, 32] the pooled proportion increased to 12.63% (95% CI, 5.84%-25.22%; I² = 81.77%). These findings support the view that patient age and cohort enrichment are major determinants of the reported frequency of *DICER1* alterations in thyroid lesions.

Assessment of small-study effects with Egger’s regression test yielded a borderline result (*p* = 0.059). Given the limited number of pooled studies and the marked clinical and methodological heterogeneity, this finding was interpreted cautiously and was not considered sufficient evidence of publication bias on its own. Overall, the quantitative analysis indicates that *DICER1* alterations are uncommon in large unselected adult thyroid cohorts but substantially enriched in pediatric, young-adult, and histotype-enriched settings.

### Qualitative clinicopathologic synthesis

Additional thyroid-focused studies were retained for qualitative clinicopathologic synthesis because they substantially refined the interpretation of *DICER1*-associated thyroid disease but did not provide a denominator suitable for prevalence meta-analysis. These studies included cytology-histology correlation series, molecularly selected *DICER1*-positive FNA cohorts, multicenter observational studies restricted to *DICER1*-mutated nodules, a multinodular goiter study centered largely on germline or synonymous variants, and a morphology-enriched study of Chinese cases [35–40]. The main features of these studies are summarized in Table 2.

Across the qualitative clinicopathologic literature, *DICER1* alterations were reported in a broad spectrum of thyroid lesions rather than in a single disease category. These included follicular nodular disease or multinodular goiter, follicular adenoma, follicular thyroid tumor of uncertain malignant potential, noninvasive follicular thyroid neoplasm with papillary-like nuclear features, follicular thyroid carcinoma, papillary thyroid carcinoma, poorly differentiated thyroid carcinoma, high-grade differentiated thyroid carcinoma, and rare primitive malignant thyroid tumors such as thyroblastoma. Cytomorphologically, these studies consistently emphasized predominantly follicular-patterned lesions with frequent macrofollicular or mixed macro-/microfollicular architecture, relatively bland or only subtly atypical nuclei, variable colloid, and frequent preoperative classification within benign or indeterminate cytologic categories. Taken together, the qualitative studies refine the clinicopathologic spectrum of *DICER1*-associated thyroid disease and provide the morphologic context required to interpret the pooled quantitative results.

## Discussion

The present review indicates that *DICER1* alterations represent a genuine but highly context-dependent feature of thyroid pathology. This conclusion emerges most clearly when the prevalence-eligible studies are interpreted individually rather than collapsed into a single undifferentiated estimate. In the largest routine-practice adult cohort, Chong et al. [28] identified *DICER1* hotspot mutations in only a small minority of adult-onset indeterminate thyroid nodules, supporting the view that *DICER1* is uncommon in broad adult molecular testing practice. Importantly, that study also showed relative mutual exclusivity between *DICER1* hotspot mutations and other thyroid driver alterations, supporting a driver-associated rather than purely incidental role. At the opposite end of the spectrum, pediatric and pediatric-enriched studies reported substantially higher frequencies. Onder et al. [29] found *DICER1* mutations in 8 of 56 pediatric papillary thyroid carcinomas and showed that these tumors were enriched in low-risk follicular-patterned papillary thyroid carcinomas, particularly invasive encapsulated follicular variant papillary thyroid carcinomas, with female predominance and absence of distant metastasis. Similarly, Bae et al. [34] identified *DICER1* as the most frequent alteration in pediatric follicular-patterned thyroid tumors, present in 9 of 41 cases, across follicular adenoma, follicular thyroid carcinoma, and NIFTP-like lesions. In the pediatric and young-adult cohort of Mastnikova et al. [32], *DICER1* variants were found in 24 of 350 nodules, and *DICER1*-positive papillary thyroid carcinomas were associated with follicular subtype, encapsulation, absence of extrathyroidal extension, and fewer lymph node metastases. Together, these quantitative studies show that the frequency of *DICER1* depends strongly on age setting and lesion enrichment, and that the overall pooled estimate should not be read as a universal prevalence figure. The remaining predominantly adult studies in the quantitative synthesis further refine this pattern. Lee et al. [17] examined 462 consecutive FNAs with available ThyroSeq testing and found only 8 *DICER1*-mutated nodules, again underscoring the rarity of these alterations in routine adult thyroid practice. However, the nodules in that study occurred in younger adult women, were larger than controls, and typically showed bland cytomorphology with abundant colloid, while available resections were benign and mostly follicular adenomas. Wang et al. [31] identified *DICER1* mutations in 52 of 899 Bethesda II/III/IV nodules and demonstrated mutual exclusivity with *BRAF*V600E in Bethesda III/IV lesions, reinforcing the concept that *DICER1*-positive lesions may occupy a distinct molecular niche rather than simply overlapping with canonical *BRAF*-driven disease. Minna et al. [30], although based on a smaller cohort, added an important bridge between adult molecular pathology and follicular-patterned tumor biology by identifying somatic *DICER1* mutations in adult follicular-patterned, *RAS*-like thyroid tumors. These findings collectively support a recurring theme across the adult literature: *DICER1* is uncommon in unselected thyroid testing populations, but when present, it tends to occur in a follicular-patterned, RAS-like clinicopathologic context rather than in classic BRAF-like papillary carcinoma.

The qualitative studies sharpen this interpretation considerably because they describe the actual morphologic phenotype of *DICER1*-associated thyroid lesions. Darbinyan et al. [35] provided one of the earliest focused cytology–histology correlation studies and showed that *DICER1*-mutated thyroid FNAs can display two broad cytologic patterns: architectural atypia with a microfollicular pattern and scant colloid, or mild nuclear atypia without overt papillary-type features. On resection, those cases included follicular carcinoma, papillary thyroid carcinoma, and poorly differentiated thyroid carcinoma, demonstrating early on that *DICER1* was not restricted to a single benign phenotype. Lee et al. then extended this concept in adult practice by showing that many *DICER1*-mutated nodules may actually appear deceptively bland and may even be interpreted as benign on retrospective review, despite their molecular profile [17]. These two studies together make an important diagnostic point: *DICER1*-associated lesions can be cytologically subtle and may not declare themselves through overtly malignant FNA features.

The most detailed clinicopathologic refinement comes from Lengyel et al. [36], who evaluated the largest FNA-based cohort of *DICER1*-altered thyroid lesions in a multipractice setting. Their study integrated cytomorphology, histology, and molecular data, showing that these lesions typically display mixed macro-/microfollicular architecture, variable cellularity, scant to moderate colloid, and only insignificant to mild nuclear atypia. Histologically, their cases ranged from follicular adenoma and minimally invasive follicular thyroid carcinoma to NIFTP and high-grade follicular-derived non-anaplastic thyroid carcinoma. Their discussion is particularly valuable because it places their own findings in direct continuity with both Darbinyan et al. and Lee et al., while also emphasizing that many *DICER1*-altered lesions are overall *RAS*-like in appearance [17, 35]. At the same time, Lengyel et al. caution that the biologic spectrum is broader than purely low-risk tumors, because high-grade follicular-derived carcinomas can also occur in the setting of *DICER1* alterations [36]. These findings support the view that *DICER1* should not be regarded as merely an incidental molecular finding.

Jitpasutham et al. add an additional layer by comparing *DICER1*-mutated and *PTEN*-altered thyroid FNAs [37]. Their cohort of 117 molecularly selected FNAs, including 36 with *DICER1* mutation, showed that *DICER1*-mutant lesions are often associated with younger age, multinodular background, and indeterminate cytology, especially AUS and follicular neoplasm categories. Histologic follow-up broadened the lesion spectrum to include adenomatous nodules or follicular adenomas, NIFTP, papillary thyroid carcinoma, and thyroblastoma. This study is especially useful because it underscores that *DICER1*-positive thyroid disease often emerges in the context of multinodularity and indeterminate follicular-patterned cytology, which is exactly the setting in which pathologists may face interpretive uncertainty.

The more recent multicenter study by Karimkhan et al. shifts the focus from morphology alone to the clinical behaviour of *DICER1*-positive Bethesda III/IV nodules [38]. Although that study cannot be used for pooled prevalence because it included only *DICER1*-mutated nodules, it is highly informative for management-oriented interpretation. The authors found a malignancy rate of 31.8% among resected *DICER1*-positive nodules, with papillary thyroid carcinoma, follicular carcinoma, and a small number of poorly differentiated carcinomas represented in the malignant group. They also confirmed that most mutations involved hotspot RNase IIIb regions and discussed the possibility that the malignancy risk of *DICER1*-positive indeterminate nodules may be higher than average, although they appropriately acknowledged the influence of additional mutations and selection bias. This study therefore suggests that *DICER1* is not a lesion-specific marker of malignancy, but may still carry clinically relevant risk in indeterminate adult thyroid nodules.

The multinodular-goiter study by Miranda et al. [39] is informative for a different reason. Unlike the oncology-oriented cohorts, this study focused on large multinodular goiters and found mostly synonymous or likely benign germline *DICER1* variants, with no compelling second-hit pattern in the large majority of cases. The study therefore should not be read as evidence of oncogenic *DICER1* lesion frequency comparable to the hotspot-driven cohorts. However, it is useful in broadening the clinicopathologic landscape of thyroid *DICER1* findings by showing that benign multinodular disease and constitutional variation can also enter the diagnostic conversation. It therefore helps justify why not every *DICER1*-related thyroid study can or should be treated as quantitatively commensurable with hotspot-driven neoplastic cohorts.

Wu et al. [40] add one more qualitative signal that is remarkably consistent with the rest of the literature. In their series, 15 of 163 morphology-enriched suspected *DICER1*-related tumors were confirmed to harbour *DICER1* hotspot mutations, predominantly D1709 and E1813. Cytologically, the authors described a predominantly macrofollicular pattern with small, uniform, round, dark nuclei, with only occasional nuclear grooves and frequent assignment to benign or indeterminate categories. Histologically, the resection diagnoses ranged from follicular thyroid nodular disease and follicular adenoma to follicular thyroid tumor of uncertain malignant potential, follicular thyroid carcinoma, and high-grade differentiated thyroid carcinoma. Even though the cohort was morphology-enriched, its findings align closely with the message emerging from Darbinyan, Lee, Lengyel, and Jitpasutham: *DICER1*-associated thyroid lesions are often follicular-patterned, cytologically bland, and distributed across a broad benign-to-malignant spectrum [17, 35–37].

Taken together, the full body of quantitative and qualitative evidence suggests that *DICER1* identifies a clinicopathologic setting, not a single entity. Quantitative prevalence studies show that *DICER1* is uncommon in broad adult routine-practice cohorts but enriched in pediatric, young-adult, and follicular-patterned settings. Qualitative studies show that these lesions often share a recurring morphologic profile (i.e., macrofollicular or mixed follicular growth, bland nuclei, variable colloid, and frequent indeterminate cytology) but may still culminate in a wide range of histologic outcomes, from benign nodular disease to high-grade carcinoma and thyroblastoma. For the practicing pathologist, the implication is clear: a *DICER1* alteration should neither be dismissed as meaningless nor overcalled as a carcinoma-defining event. Its value lies in integrated interpretation with morphology, lesion type, age, and clinical context.

This integrative approach is particularly important in younger patients, as the literature consistently shows that pediatric and young-adult cases are enriched for *DICER1* alterations and more often carry constitutional implications [29, 32, 34]. At the same time, the malignant phenotype in these younger cohorts often remains relatively indolent and follicular-patterned rather than overtly aggressive. Accordingly, the practical significance of a thyroid *DICER1* alteration in a child or young adult extends beyond local tumor classification: it may also represent an entry point to broader consideration of *DICER1*-related tumor predisposition, especially in the presence of multinodular disease, family history, or additional syndromic clues [29, 32, 34].

The broader biologic and constitutional literature also supports this interpretation. Mechanistic studies have shown that *DICER1* hotspot mutations, particularly within the RNase IIIb domain, impair microRNA processing in a selective rather than complete-loss-of-function manner, thereby creating a biologically distinctive context rather than a conventional single-pathway driver state [41]. Structural studies of the human DICER–pre-miRNA complex further clarify how cancer-associated mutations may perturb RNA recognition and cleavage, whereas germline-oriented studies support the view that thyroid disease may represent one of the most frequent and sometimes earliest manifestations of *DICER1*-related tumor predisposition [15, 42–44] These data provide a biologic framework for understanding why *DICER1*-associated thyroid lesions can span a broad morphologic spectrum while remaining unified by a shared molecular background.

These study-level findings also justify the analytic framework adopted in the present review. Prevalence-oriented studies and clinicopathologically enriched series address related, but non-equivalent, questions: the former estimate how frequently *DICER1* alterations are reported in defined thyroid cohorts, whereas the latter characterize the morphologic and clinicopathologic spectrum of *DICER1*-associated thyroid lesions. Their separate consideration is therefore a strength of the review, as it avoids pooling fundamentally different study designs into a single summary estimate that could be methodologically neat but biologically misleading. This distinction is critical to a robust and clinicopathologically meaningful interpretation of the current thyroid *DICER1* literature.

Overall, these findings indicate that the relevance of *DICER1* in thyroid pathology lies less in defining a single diagnostic category than in recognizing a biologically coherent, morphologically diverse subset of thyroid lesions best interpreted through an integrated clinicopathologic and molecular approach.

## Conclusions

In conclusion, *DICER1* alterations are uncommon in large unselected adult thyroid cohorts, but are reproducibly enriched in pediatric and young-adult patients and in follicular-patterned thyroid lesions. The contemporary literature indicates that these alterations occur across a wide clinicopathologic spectrum, encompassing multinodular and follicular nodular disease, follicular adenoma, NIFTP, follicular thyroid carcinoma, follicular-patterned papillary thyroid carcinoma, and, more rarely, high-grade or primitive malignant tumors such as thyroblastoma. Accordingly, *DICER1* is best understood not as a lesion-specific marker, but as a molecular finding whose diagnostic significance depends on integration with morphology, lesion type, patient age, and clinical context.

This review also highlights that a methodologically sound interpretation of the field requires separate consideration of prevalence-eligible cohorts and enriched clinicopathologic series. Under this framework, the quantitative prevalence of *DICER1* alterations remains limited overall, yet their biological and diagnostic relevance becomes more apparent. Specifically, *DICER1* identifies a clinically meaningful subset of thyroid lesions, particularly within the follicular-patterned spectrum, rather than defining a single nosologic entity. In younger patients, especially those with multinodular thyroid disease or a suggestive personal or family history, its detection may also serve as an important clue to underlying *DICER1*-related tumor predisposition. Further studies using standardized molecular criteria, systematic germline investigation, and integrated cytology-histology correlation are needed to better define the diagnostic and clinical implications of *DICER1* in thyroid pathology.

## Electronic Supplementary Material

Below is the link to the electronic supplementary material.

Supplementary Material 1 **Supplementary Figure 1a. PRISMA flow diagram of literature selection**. A total of 163 records were identified from PubMed, Scopus, and Web of Science. After duplicate removal, 101 records were screened and 26 full-text articles were assessed for eligibility. Thirteen studies met the inclusion criteria for the systematic review; of these, seven were included in the quantitative synthesis and six in the qualitative clinicopathologic synthesis only. Reasons for full-text exclusion are detailed in the figure (JPG 124 KB)

## Data Availability

The raw materials underlying this study are available from the corresponding author upon reasonable request.
