# Supplementary figures and images for: DICER1 alterations in thyroid lesions: a systematic review and meta-analysis with clinicopathologic implications
Source: Virchows Arch. 2026 May 28;489(1):3–14. doi: 10.1007/s00428-026-04580-5 (PMC13369623; doi:10.1007/s00428-026-04580-5)

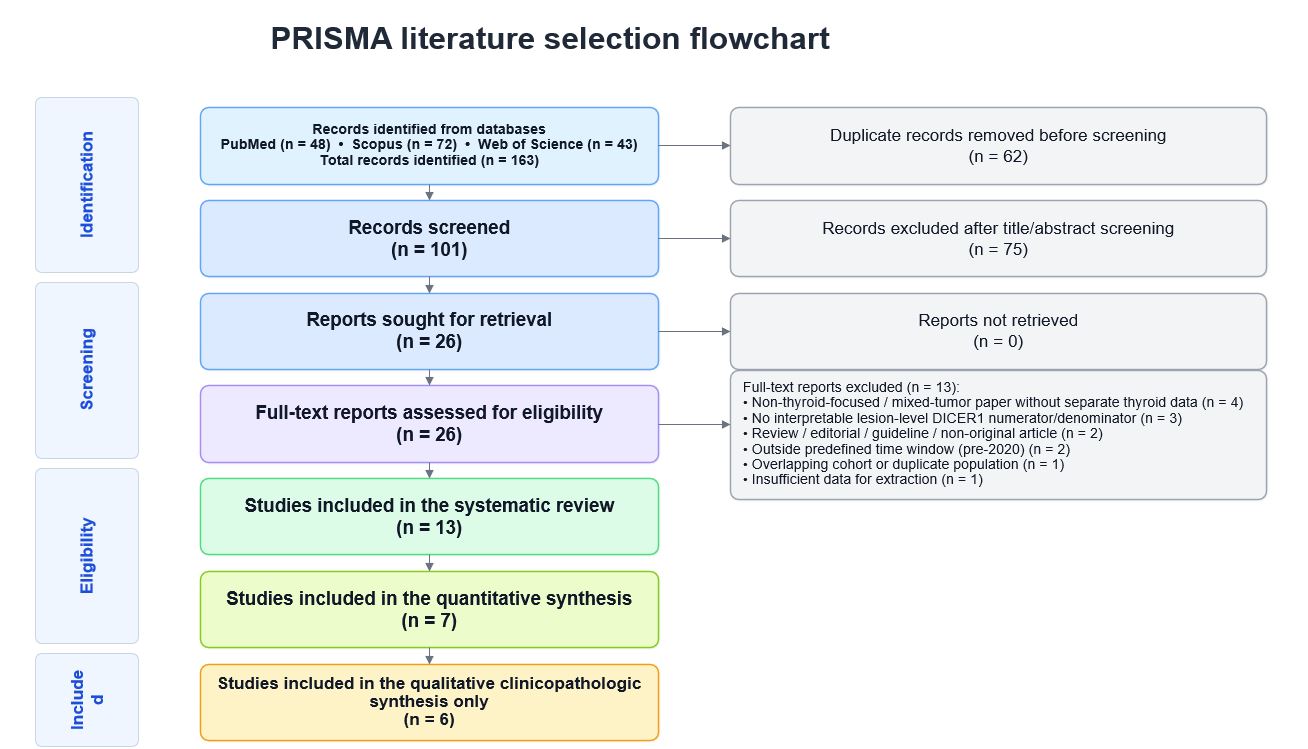

Supplement: Supplementary file 1 — Supplementary Material 1 Supplementary Figure 1a. PRISMA flow diagram of literature selection. A total of 163 records were identified from PubMed, Scopus, and Web of Science. After duplicate removal, 101 records were screened and 26 full-text articles were assessed for eligibility. Thirteen studies met the inclusion criteria for the systematic review; of these, seven were included in the quantitative synthesis and six in the qualitative clinicopathologic synthesis only. Reasons for full-text exclusion are detailed in the figure (JPG 124 KB) [file 428_2026_4580_MOESM1_ESM.jpg]
